# Critical Reliability Issues of Common Type Alcohol-Based Handrub Dispensers

**DOI:** 10.1186/s13756-020-00735-4

**Published:** 2020-06-22

**Authors:** Száva Bánsághi, Hervé Soule, Chloé Guitart, Didier Pittet, Tamás Haidegger

**Affiliations:** 1 Department of Epidemiology, Semmelweis University, Budapest, Hungary; 2 University of Geneva Hospitals and Faculty of Medicine (HUG), Geneva, Switzerland; 3 University Research and Innovation Center (EKIK), Óbuda University, Budapest, Hungary; 4 Austrian Center for Medical Innovation and Technology (ACMIT), Wiener Neustadt, Austria

**Keywords:** Hand hygiene, Handrub volume, Dispenser quality, Alcohol-based handrub, Handrub dosing, Patient safety standards, Hand sanitizer

## Abstract

**Background:**

Hand hygiene can only be efficient if the whole hand surface is treated with sufficient alcohol-based handrub (ABHR); therefore, the volume of handrub applied is a critical factor in patient safety. The proper amount of ABHR should be provided by handrub dispensers. The aim of this study was to investigate the dispensing performance of wall-mounted ABHR dispensers commonly employed in hospital settings.

**Method:**

In a multicenter study, we tested 46 dispensers (22 in laboratory and 24 in clinical environments), measuring dispensed ABHR volume during continuous use and after a period of non-use. The influence of the pumping mechanism, liquid level, ABHR formats, handrub composition, temperature, and atmospheric pressure was investigated.

**Results:**

A total of 7 out of the 22 investigated dispensers (32%) lost a significant amount of handrub; greater than 30% of the nominal volume after 8 h of non-use, thus frequently dispensing suboptimal volume, as measured in laboratory settings. Key influencing factors were found to be handrub format (gel or liquid), handrub level in the container and type of dispenser. When gel ABHR was used, after 4 h of non-use of the dispensers, the volume of the dispensed amount of ABHR insignificantly changed (97% of the original amount), while it technically decreased to zero in the case of liquid ABHR (1% of the original amount). The liquid level had a medium effect on the dispensed volume in each investigated case; the magnitude of this effect varied widely depending on the dispensing mechanism. When dispensers were in continuous use, they dispensed a cumulated 3 mL of ABHR from two consecutive pushes, while when they were not in use for 1 h, up to 4 consecutive pushes were necessary to provide a total of 3 mL ABHR. Design and production quality were also identified as important contributing factors with respect to the volume dispensed. Data collected in clinical settings confirmed these findings, for multiple types of dispensers.

**Conclusion:**

All ABHR dispensers should be regularly audited to control the reference volume distributed, with particular attention paid to regular mechanical pump units filled with liquid handrub.

## Introduction

Hand hygiene is the most important measure to prevent healthcare-associated infections, slow down major epidemics and prevent the spread of antimicrobial resistance [1]. In the last 20 years, performing hand hygiene with alcohol-based handrub (ABHR) has become globally accepted [2]. Handrubbing has several advantages compared to soap and water handwashing; it acts faster, being more effective and better tolerated by skin, and can always be performed at the point of patient care. In 2015, ABHR was included in the World Health Organization’s (WHO) Essential Medicines List [3].

Handrubbing is only effective if the whole hand surface is covered with an adequate volume of ABHR. However, the healthcare workers (HCWs) performance is variable. In a study about hand hygiene performance, 5200 clinical staff members were investigated, and only 72% of them reached acceptable hand coverage with ABHR [4]. Another study investigated 1269 HCWs; only 67% covered their hands properly when assessed by a fluorescent method [5].

Both the EN 1500 European Norm (test method to evaluate the efficacy of a handrub) and the North American standard ASTM E−1174 require the application of twice 3 ml of handrub [6, 7]. Since 2006, the WHO recommends performing hand hygiene using a “palmful” amount of handrub, considering that the volume of handrub to use varies with the size of the HCW’s hands [2]. A study showed that at least 2 mL of ABHR is needed to completely cover all hand surfaces, but 3 mL may be insufficient in the case of large hands; definitely, a volume of 1 mL of ABHR cannot cover the entire hand surface [8]. The volume of handrub applied plays a critical role in bacterial reduction following handrubbing [9]. Another study demonstrated that a higher bacterial load reduction was reached by applying more handrub: using 1.1 mL, 2 mL or 5 mL of the same ABHR resulted in 1.85, 3.35, and 3.58 log_10_ reduction, respectively [10].

HCWs can rarely quantify the dispensed amount of ABHR used in daily clinical duties. If ABHR dispensers aliquot insufficient amount of handrub, individuals will remain unaware that the reduced amount of ABHR may lead to reduced effectiveness, and that can increase the risk of cross-transmission.

**Fig. 1:**
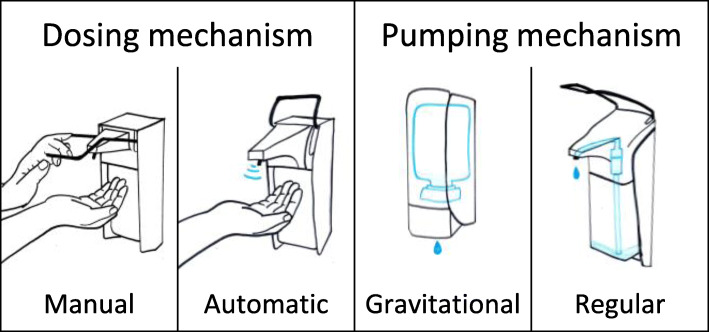
Classification of wall-mounted handrub dispensers by dosing and pumping mechanism

**Fig. 2:**
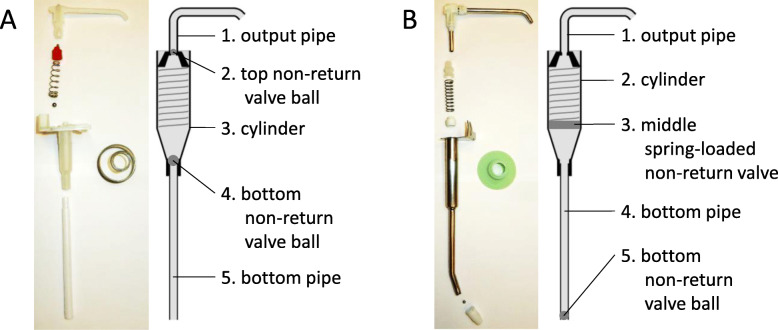
Structure of two different pumps; both from regular, manual dispensers

ABHR might be provided from a wide range of dispensers: individual bottles, disposable plastic bottles or wall-mounted systems. A majority of hospitals use wall-mounted dispensers. Based on the dosing mechanism, two main categories of the wall-mounted dispensers exist; manual and automatic (Fig. 1). With respect to pumping, wall-mounted dispensers are equipped with either regular or gravitational pumping mechanisms. Gravitational dispensers have a valve mechanism at the bottom of the bottle/bag, so that the handrub can flow out upon dispensing. In contrast, regular dispensers operate with a bottle of ABHR enclosed, with a pumping mechanism to lift the handrub when applied. The pumps have a certain kind of closure system to hold the ABHR. Figure 2 shows the construction of two different regular pumps. A common advantage of regular dispensers is that universal size ABHR bottles can be used, unlike gravitational dispensers that typically work with custom-designed refills.

The objective of the current study was to comprehensively investigate the reliability of different types of wall-mounted ABHR dispensers used in hospitals.

## Methods

### Definitions

Important definitions are given in Table 1.

**Table 1:** Important definitions collected to improve the readability of the article

ABHR formats	ABHR exists on the market in foam, gel or liquid format.
ABHR composition	ABHRs contain ethanol, propanol, isopropanol or a combination of these as active ingredients, and may contain additional active ingredients.
Dispensed volume	The measured volume of ABHR that a dispenser actually aliquot.
Baseline volume	The *dispensed volume* aliquoted by the dispenser after 5 consecutive strokes or applications.
Volume loss	The difference between the *baseline volume* and the *dispensed volume* after a dedicated time of non-use (letting the dispenser to rest idle).
Adjusted volume	The intended volume that a dispenser should dispense (based on the user guide / manufacturer’s description) when operated it a single time (pulling the arm of the dispenser once, or in case of automatic dispenser activate the sensor once). Some dispensers can only be used with fixed nominal volume, other may provide the option to adjust the target nominal volume to be dispensed.
Accuracy	The difference between the *adjusted volume* and the *dispensed volume*.
Performance	The overall capability of a dispenser to operate according to its specification, including its time-dependent *accuracy*.

### Setting

We conducted a multicenter study testing key parameters possibly associated with the performance of wall-mounted ABHR dispensers. Most of the data were collected in laboratory environments, in a private research laboratory in Hungary and at the University of Geneva Hospitals (HUG), Switzerland. In addition, selected dispensers were investigated under clinical conditions at South-Pest Hospital Centre, Hungary and at the National Koranyi Institute of TB and Pulmonology, Hungary to confirm laboratory findings. For each parameter and environmental condition tested, an adequate assessment method was chosen, as described below.

### Hanrub dispensers

In laboratory settings, we evaluated a total of 22 commercially available, wall-mounted ABHR dispensers. Five dispensers were in actual clinical use before, while 17 were new and purchased for the purpose of the study. Most of the dispensers were manual (18), while 4 were automatic. We tested both regular (17) and gravitational (5) dispensers. In a majority of dispensers (15), the volume was adjustable. Whenever possible, the volume was set at 1.5 mL; otherwise it was set at 2 mL. Table 2 lists the exact type of the investigated dispensers, along with their manufacturers.

**Table 2:** Wall-mounted dispensers involved in the laboratory investigation

Manufacturer*	Type of dispenser
BODE Chemie GmbH (Hamburg, Germany)	Eurospender 1 plus
	Eurodispenser Vario (both 500 mL and 1000 mL capacity)
	Dispenser for Sterilium Gel
Gojo Industries Inc. (Akron, OH, USA)	Purell ADX-7
	Purell LTX-12
	Purell TFX
Ophardt Hygiene-Technik GmbH (Issum, Germany)	ER-T
	Ingo-man 26
	Different Ingo-Man Plus types: ELS A/K, TLS A/24 – both 500 and 1000 mL, TLS P/24, T/TLS, IMP with counter (2pcs)
Schülke & Mayr GmbH (Norderstedt, Germany)	Schülke D1 Touchless
	Schülke SM2 (2pcs)
	Short lever dispenser (2pcs)
Soaptronic International LLC (Lake Forest, CA, USA)	Germstar no-touch dispenser

*listed in alphabetical order

### Measuring the volume of handrub dispensed

After each sampling, dispensers were pressed at least 5 times to make sure that the pump was at an actual new baseline.

The weight of the dispensed amount of ABHR was measured by analytical balance (Sartorius Analytic, AC 210 P, Sartorius GmbH, Göttingen, Germany). For calibration, 1 mL of each liquid ABHR was weighted, dosed with an automatic pipette (Eppendorf Research Plus, Eppendorf AG, Hamburg, Germany). The average value of 10 measurements was used to convert the measured weight of liquid ABHR to volume.

For gels, it was not possible to measure precisely 1 mL of the product using the automatic pipette; therefore, the official density value was used from the safety data sheet of the product manufacturer for converting weight to volume; alternatively, we indicated the weight instead of the volume of the product below.

For the measurements in the clinical settings, the official density value was used for all hand hygiene products.

### Data analysis

Series of parallel measurements were performed and are indicated for each experimental parameter. Measurements were repeated to test the variability of the data. The value of a data point was calculated as the mean of concomitant measurements + standard deviation (SD). Data analysis was carried out using Microsoft Excel 2016.

### Time-dependency

All 22 dispensers were tested for the basic hypothesis that they would lose significant volume (dispensed volume compare to the baseline volume) after several defined time period of non-use. The maximum non-use duration tested was 12 h, corresponding to the situation in a healthcare facility, in the absence of night shifts.

- Dispensers included: all
- Number of parallel measurements for each variable parameter: 5. For dispensers measured at HUG (#G, #H, #I, #J, #K, and #P), 2–3 parallel values were measured.
- Handrub used: dispensers with universal Euro-bottle were filled with liquid ABHR. For custom refill dispensers, their own (proprietary) refill ABHR was used. Dispensers #M, #Q, and #T were tested using gel ABHR; liquid ABHR was used for all others.
- Time period of non-use before measurements: 0, 1, 2, 4, 8 and 12 h.
- Liquid level in the container:
  - If the dispenser had refillable container: 300–400 mL for 1 L bottles, and 150–200 mL for 500 mL bottles
  - If not: handrub level was not controlled.

### Liquid level

To test the effect of the liquid level in the bottle/container on the dispensed volume, the dispensed volume was measured while the amount of handrub in the bottle was controlled. Experiments were carried out using dispensers that had been found to lose volume.

- Units included: Dispenser #A, #B and #F
- Number of parallel measurements for each variable parameter: 5
- Handrub format: liquid
- Handrub composition: 70% ethanol and 2.5% glycerin
- Time period of non-use before measurements: 0, 15, 30, 45, 60 min
- Liquid level in the container: 100–200 mL, 300–400 mL, 500–600 mL, 700–800 mL and 900–1000 mL

### Pumping mechanism

Experiments were conducted to determine how the dispenser returns to normal performance after non-use. To better investigate their pumping mechanism, after a period of non-use, the first five doses of handrub were collected separately, and each was weighted. To further investigate the pumping mechanism, selected dispensers were taken apart, and their structure studied by research engineers from Óbuda University.

- Units included: Dispenser #A, #B and #E
- Liquid level in the container: 300–400 mL
- Time period of non-use before measurements: 1, 2, 4 and 8 h

### Handrub format

During the following experiments, the aim was to identify key contributing factors to the time-dependency. Different ABHR formats – gel and liquid – were tested on the same dispenser that had been found to lose a significant amount of handrub during a short period. In one case, we also included liquid soap, as these dispensers are also suitable, and wildly used for dispensing soaps. Experiments were repeated with different handrubs and different dispensers.

#### First experiment

- Units included: Dispenser #A
- Number of parallel measurements for each variable parameter: 5
- Time period of non-use before measurements: 0, 1 and 2 h
- Handrub format:
  - Liquid: Semmelweis Rub (Molar Chemicals Ltd.)
  - Gel: BradoLife Gel (Florin Zrt)
  - Soap: Brado Disinfectant Liquid Soap (Florin Zrt)
- Liquid level in the container: 300–400 mL

***Second experiment***

- Dispenser included: dispenser #A
- Number of parallel measurements for each variable parameter: 10
- Time period of non-use before measurements: 0, 1, 2, 4, 8 and 12 h
- Handrub format:
  - Liquid: Sterillium Classic Pure (BODE Chemie GmbH)
  - Gel: Sterillium Gel (BODE Chemie GmbH)
- Liquid level in the container: 300–400 mL

***Third experiment***

- Dispenser included: Dispenser #G
- Number of parallel measurements for each variable parameter: 2–3
- Time period of non-use before measurements: 0, 1, 2, 4, 8 and 12 h
- Handrub format:
  - Liquid: Hopirub (B Braun Medical AG)
  - Gel: Sterillium Gel (BODE Chemie GmbH)
- Liquid level in the container: 300–400 mL

### Compositions of liquid handrubs

As prior experiments showed that the volume-loss effect was insignificant with gel handrubs, only liquid handrubs were tested in the follow-up experiments. Seven different commercially available liquid handrubs with different compositions were tested on the same dispenser that previously proved to loose a significant amount of handrub within a short period of time.

- Unit included: Dispenser #F
- Number of parallel measurements for each variable parameter: 10
- Liquid level in the container: 300–400 mL
- Time period of non-use before measurements: 0, 15, 30, 45 and 60 min
- Liquid handrubs tested:
  - Promanum Pure, B Braun Medical AG (73.4% ethanol, 10% propan-2-ol)
  - Softa-Man, B Braun Medical AG (45% ethanol, 18% propan-1-ol)
  - Manusept basic, BODE Chemie GmbH (80% ethanol)
  - Sterillium, BODE Chemie GmbH (30% propan-1-ol, 45% propan-2-ol, 0.2% mecetronium ethylsulfate)
  - Semmelweis Rub, Molar Chemicals (70% ethanol).
  - Desderman Pure, Schülke & Mayr GmbH (78.2% ethanol, 0.1 g biphenyl-2-ol)
  - Desmanol Pure, Schülke & Mayr GmbH (75% ethanol, < 1% myristylalcohol)

### Temperature and atmospheric pressure

For the temperature experiment, Dispenser #A was selected, as this dispenser showed the most significant leaking effect. The dispenser was placed in a small room, and the temperature of the room was changed between 22 °C and 28 °C. The temperature range was chosen to be narrow because only room temperature is relevant in clinical settings. A hole was drilled to the side of the liquid container, and a thermometer was placed into the hole, reaching into the handrub solution. For every data point, the handrub’s temperature was recorded instead of the room temperature.

In further experiments, the atmospheric pressure values were also recorded. We used data from a nearby meteorological station that published the atmospheric pressure data to a public website, refreshing every minute.

- Unit included: Dispenser #A
- Liquid level in the container: 100–200 mL
- Time period of non-use before measurements: 15 min

### Accuracy of dispensing

To determine the accuracy of dispensers, i.e., the dispensed amount versus the adjusted volume, three dispensers were selected, where at least 3 different dispensing volumes could be adjusted. One of these dispensers allowed for the volume to be modified continuously with a turning knob. One potential source of measurement error was to set the volume incorrectly. To avoid this error, only five measurements were taken at every preset volume, before the dial would be adjusted to another volume. After being readjusted to the target volume, another five measurements were taken, until a total of 20 measurements were collected.

- Handrub format: liquid
- Number of measurements for each variable parameter: 20 parallel
- Units included: Dispenser #F, #O #S
- Adjusted volume:
  - Dispenser #F: 0.75 mL, 1 mL, 1.2 mL, 1.5 mL
  - Dispenser #O: 1 mL, 2 mL, 3 mL
  - Dispenser #S: 0.7 mL, 1 mL, 1.5 mL, 3 mL

### Relevance in clinical settings

To demonstrate that our findings on the volume-loss phenomenon have an actual clinical impact, some of the measurements were repeated in acute care hospitals. To reduce the disruption to the hospital’s procedures, dispensers were investigated within a shortened period of time, and the number of repeated measurements was also decreased.

To demonstrate that ABHR format has an important role in volume-loss, in situ data were collected from dispensers in the surgical ward of a local hospital. Dispensers were located on the wall right in front of the operating room and were in every-day clinical use. The measured dispensers were of the same type (manual, regular, with steel arm), as far as we could investigate, they were installed at the same time, and were filled with three different products; a liquid handrub, a gel handrub and a handwashing lotion.

- Time period of non-use before measurements: 0, 15 and 30 min
- Number of parallel measurements for each variable parameter: 3
- Dispensers were filled with:
  - Liquid handrub: Sterillium Classic Pure (BODE Chemie GmbH)
  - Gel handrub: Aniosgel 85 NPC (Laboratoires Anios)
  - Washing lotion: Baktolin wash (BODE Chemie GmbH)

To estimate how common the volume-loss issue may be, we tested 21 wall-mounted dispensers in a hospital. Dispensers were located on the corridor, in patient rooms, in examination rooms, and in the HCWs’ room. Type of dispenser and hand hygiene product were recorded.

- Time period of non-use before measurements: 10 min
- Number of parallel measurements for each variable parameter: 3

## Results

### Time-dependency

**Fig. 3:**
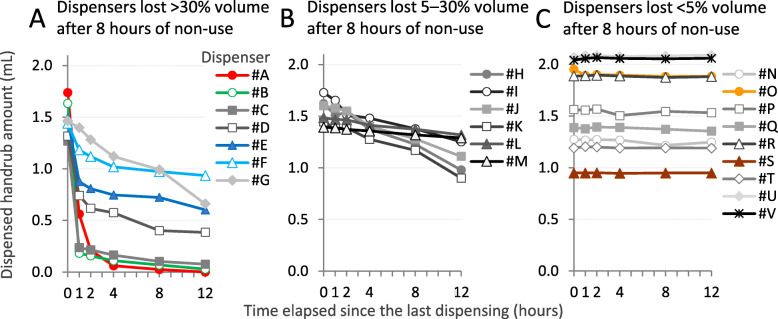
Decrease of dispensed handrub volume over time. Values are mean ± SD of *n* = 5 repeated measures, except for dispenser #G, #H, #I, #J, #K and #P (n = 2–3). Dispensers labeled with colorful marks will be also shown in later figures, using the same colors

A total of 7/22 (32%) investigated dispensers demonstrated a significant loss of dispensed volume, greater than 30% after 8 h of non-use (Fig. 3a). The dispensed volume of 9 dispensers (41%) did not vary significantly over time in any of the examined resting periods, accounting for a volume loss smaller than 5% in 8 h and even after 12 h non-use (Fig. 3c). Overall, 7/22 (32%) dispensers lost significant volume (> 5%), even over a short, 1-h period. Volume loss after 8 h of non-use was calculated for each dispenser and presented in Table 3 together with their characteristics. The worst-performing dispensers were all manual, regular types, but it is important to note that 4/7 top-performing dispensers were also regular, manual ones. Note that 8/10 worst-performing dispensers were new ones, therefore it cannot be claimed that dispensers work poorly as a result of wearing or aging.

**Table 3:** Key characteristics of the investigated dispensers and handrub volume-loss in 8 h, relative to the baseline volume (*N* = 22)

Code name	Dosing	Pumping mechanism	Condition	Volume loss in 8 h
Dispenser #A	Manual	Regular	New	99%
Dispenser #B	Manual	Regular	New	96%
Dispenser #C	Manual	Regular	New	92%
Dispenser #D	Manual	Regular	New	70%
Dispenser #E	Manual	Regular	Used	52%
Dispenser #F	Manual	Regular	Used	32%
Dispenser #G	Manual	Regular	New	32%
Dispenser #H	Manual	Regular	New	24%
Dispenser #I	Manual	Regular	New	20%
Dispenser #J	Manual	Regular	New	20%
Dispenser #K	Manual	Regular	New	19%
Dispenser #L	Manual	Regular	New	7%
Dispenser #M	Automatic	Gravitational	New	5%
Dispenser #N	Automatic	Gravitational	New	5%
Dispenser #O	Manual	Regular	Used	3%
Dispenser #P	Manual	Regular	New	1%
Dispenser #Q	Manual	Gravitational	New	1%
Dispenser #R	Manual	Regular	New	1%
Dispenser #S	Automatic	Gravitational	Used	0%
Dispenser #T	Automatic	Gravitational	New	0%
Dispenser #U	Manual	Regular	New	-1%
Dispenser #V	Manual	Regular	Used	-1%

Footnote to the Table: Volume loss (average of 5 measures) at 8 h are indicated as percentages. Negative percentages indicate that the dispenser actually distributed more handrub than measured at baseline; such 1% difference can be explained by the variability induced by the method

### Liquid level

**Fig. 4:**
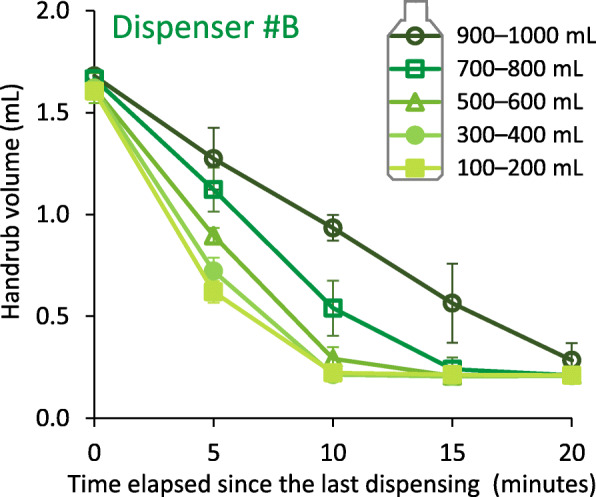
Effect of liquid level in the dispenser on the volume of dispensed alcohol-based handrub over time. Values are mean ± SD in 5 repeated measures

**Fig. 5:**
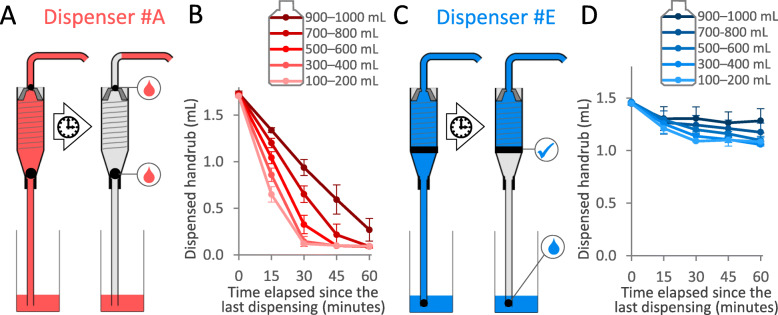
Working mechanism of two alcohol-based handrub dispensers. **a**) In the case of Dispenser #A, both of the two non-return valve ball leaked, the pump seeped almost the entire amount of stored ABHR during non-use. **b**) Effect of the handrub level in the dispenser tank for Dispenser #A. **c**) In the case of Dispenser #E, the middle spring-loaded non-return valve worked properly, while the bottom non-return valve ball did not. **d**) Effect of the handrub level in the dispenser tank in for Dispenser #E. Values in (B) and (D) are mean ± SD in 5 repeated measures

The level of liquid ABHR in the container of the dispenser proved to have a significant effect on the dispensed volume: the lower the volume, the larger the volume-loss observed. Figure 4 shows the effect of liquid level on the dispensed volume in case of Dispenser #B. Same measures were carried out with Dispenser #A and #E and displayed in Fig. 5. Note that all other experiments were carried out with controlled handrub levels.

### Pumping mechanism

**Fig. 6:**
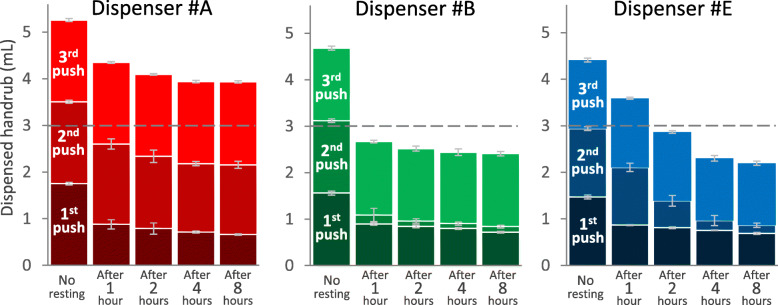
Alcohol-based handrub volumes dispensed following the first three pushes from different dispensers before and after the dispensers were not used for various time. Values are mean ± SD in 10 repeated measures

In this experiment, three different dispensers were investigated. During continuous application, all of them dispensed around 1.5 mL. Based on 80 parallel measurements, the dispensed ABHR volume averaged (mean ± SD) 1.75 ± 0.3 mL, 1.56 ± 0.04 mL and 1.47 ± 0.04 mL for Dispensers #A, #B and #E, respectively. After the dispensers were non-used for a longer period, the dispensed volume decreased; the longer the idle period, the more significant the volume-loss. We observed that the different dispensers behaved differently. In the case of Dispenser #A, only the first dose of ABHR dispensed decreased after the non-use period; then the dispenser operated normally again, aliquoting the baseline volume. In contrast, for Dispensers #B and #E, the second dose was even smaller. After the non-use period, the first five doses were measured. Every dispenser returned to normal operation (providing the baseline volume) after a maximum of three applications; therefore, only volumes of the first three pushes after the non-use period are presented in Fig. 6.

This phenomenon can be explained by the dispensers’ pumping mechanisms. Figure 5 illustrates different pumping mechanisms, differently constructed pumps, and how they work. (Note that the pumps in Fig. 5 are the same as those previously described in Fig. 2) Dispenser #A is equipped with two closures; the two valve balls should prevent the handrub from tickling back (Fig. 5a). When Dispenser #A was disassembled, we observed that none of the valves worked properly, thus almost the whole internal compartment of the pump was depleted of ABHR. Handrub only remained in the horizontal part of the output pipe. When the dispenser was used after a non-use period, the handrub had to refill the compartment from the actual handrub level. That is why the volume of dispensed handrub strongly depends on the handrub level (Fig. 5b). The dispensed amount did not decrease to zero; this probably represents the amount of ABHR that remained in the horizontal section of the output pipe. In contrast, Dispenser #E contains two stopping mechanisms: a valve and a bottom ball (Fig. 5c). We observed that the valve worked fine, while the bottom ball slowly drippled the handrub. When this type of dispenser is not in use, the handrub from the bottom part of the cylinder could tickle back to the actual handrub level, but the correctly-working valve keeps the handrub, thus a reserve of handrub remains in the upper part. During the first press, the dispenser provides the volume of handrub contained in this compartment. That is why the liquid level has a smaller impact on the dispensed amount (Fig. 5d); the volume of the first dose came mostly from this reservoir compartment. As shown, although the first dose was only slightly reduced in volume, the second dose was much less (Fig. 6).

### Handrub formats

**Fig. 7:**
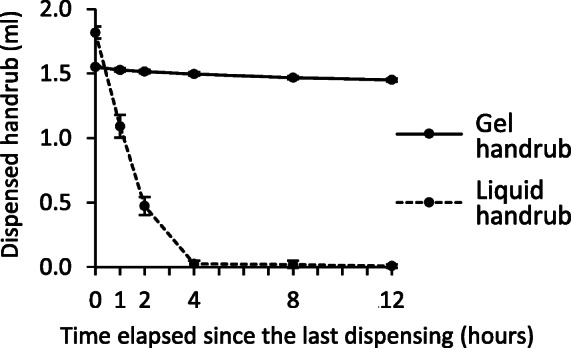
Changes of dispensed volume of handrub over time using the same dispenser (Dispenser #A) filled with liquid or gel alcohol-based handrub formats. Values are mean ± SD in 10 repeated measures

Using Dispenser #A, 10 parallel measurements were carried out at 6 different time points. While volume-loss was not significant when gel handrub was used, the dispensed volume of liquid handrub decreased significantly over time (Fig. 7). After 12 h of non-use, the dispensed volume was 0.4% versus 93.6% when the dispenser was filled with a liquid or a gel ABHR, respectively. Notably, the volume of the dispensed liquid handrub was already down near 0 mL after only 4 h. Experiments were repeated using other dispensers and other handrubs (data not shown). Results were consistent: while dispensers worked stable with gels and other more viscous hand hygiene agents, they lost volume when filled with a liquid ABHR. At baseline, when the dispensers were pressed repeatedly, every dispenser dosed more liquid handrub than gel, possibly because it revealed harder to force the gel to get dosed.

### Composition of liquid handrubs

**Fig. 8:**
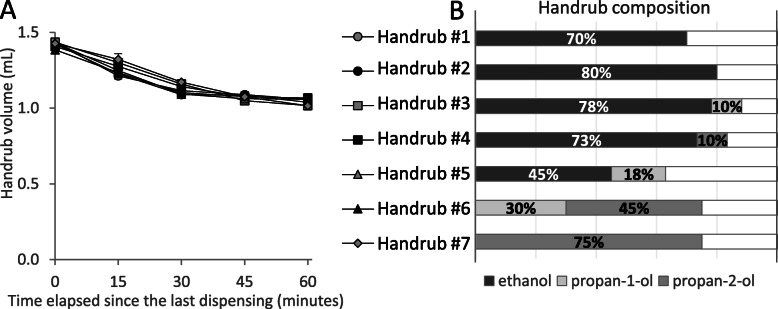
Effect of the composition of liquid alcohol-based handrubs (ABHRs) on the dispensed volume. **a**) Same dispenser (Dispenser #F) was filled with different liquid ABHRs. **b**) Composition of the investigated handrubs. Values are mean + SD in 10 repeated measures

The composition of liquid handrubs had no significant effect on the dispensed volume, the dispenser showed the same time-dependent volume loss effect when it was filled with different liquid ABHRs (Fig. 8). Together with the previous data it suggests that the viscosity of the hand hygiene product rather that its composition matters.

### Temperature, atmospheric pressure

Temperature had no significant effect on the dispensed volume within the range relevant to clinical settings. Atmospheric pressure had no effect on the dispensed volume.

### Accuracy of dispensing

**Fig. 9:**
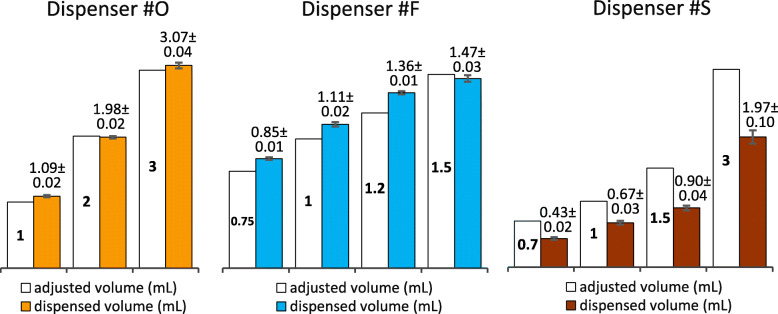
Differences between adjusted and actually dispensed volumes in case of 3 wall-mounted alcohol-based handrub dispensers. Values are mean ± SD in 20 repeated measures

The adjusted and the actually dispensed volume were compared in the case of three dispensers (Fig. 9). The adjusted and dispensed volume were technically equal for Dispenser #O. Dispenser #F showed larger differences with adjusted and dispensed volumes within a ± 15% range. For Dispenser #S, dispensed volumes were significantly lower than the adjusted volumes: 62, 67, 60 and 66%, for 0.7, 1, 1.5 and 3 mL, respectively. Dispenser #S was a gravitational dispenser, while Dispenser #O and #F were regular ones.

### Relevance in clinical settings

Dispensers used in a hospital ward were tested to assess whether the volume-loss phenomenon existed in clinical settings.

**Fig. 10:**
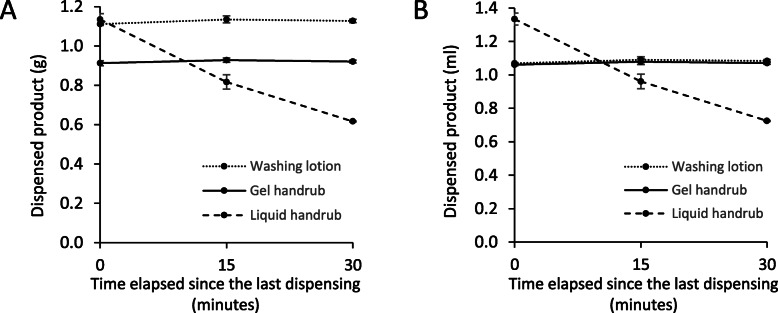
Volume-loss effect measured on the same type of dispensers, filled with different hand hygiene agents, in daily clinical use by the operating room. Weight (**a**) and volume (**b**) of the dispensed amount are presented. Values are mean ± SD in at least 3 repeated measures

Dispensers were tested in a surgical ward, at the entrance of the operating room. All the dispensers were of the same type and were filled with three different hand hygiene agents. As Fig. 10 shows, dispenser filled with liquid handrub, significant volume-loss was recorded within a short period of time, already after 15 and 30 min. Dispensers filled either with gel ABHR or with a handwashing lotion dispensed the same amount of product even after resting times of 15 and 30 min of non-use. These results confirmed prior results observed in the laboratory (Fig. 7). Note that at zero time point the weight of the dispensed gel ABHR was smaller than the weight of the other two products. It also demonstrates that gels are harder to pump due to their viscosity, therefore dispensers aliquot a smaller amount at baseline.

In another hospital, 21 dispensers were investigated. Table 4 shows 5 of them, all from the same type. Dispensers were installed at the same point in time, a few years ago, and none was reported broken or thought to work improperly as per the hospital staff. Dispensers were filled with different products; with gel ABHR in one case (Schülke Esemtan), and with liquid ABHRs in other cases. All dispensers were adjusted to 3 mL. Dispensed volume during continuous use (baseline volumes) were very different; two dispensers aliquoted almost exactly 3 mL while two others dispensed only around 60% of the adjusted volume. In 3 out of the 5 cases, the dispensed volume significantly decreased after 10 min of non-use; in 2 cases the volume-loss was less than 20%, and in one case, it was more than 50%.

**Table 4:** Accuracy and volume-loss effect in case of same-type dispensers in a hospital. ^*1^: Accuracy: adjusted vs. dispensed volume, ^*2^: Volume loss: Dispensed volume during continuous use vs. dispensed volume after 10 min of non-use

Dispenser	Adjusted volume (mL)	Hand hygiene product	Continuous use		After 10 min	
			Baseline volume (mL)	Accuracy *^1^	Dispensed volume (mL)	Volume loss *^2^
#1	3	BODE Sterillium	1.20 ± 0.01	−60%	0.51 ± 0.41	58%
#2	3	Schülke Esemtan	3.06 ± 0.01	2%	3.07 ± 0.02	0%
#3	3	BBraun Promanum	1.24 ± 0.04	− 59%	1.01 ± 0.11	19%
#4	3	BODE Manusept	3.08 ± 0.03	3%	3.02 ± 0.02	2%
#5	3	Florin Bradoderm	2.13 ± 0.63	− 29%	1.83 ± 0.76	14%

## Discussion

Our study reveals that the large majority of ABHR dispensers used in clinical settings may fail to deliver the expected volume of active handrub to appropriately cover hands. Volume-loss over time proved to be a common problem: in laboratory settings, 32% of the investigated dispensers suffered at least 30% loss of dispensed volume after 8 h of non-use. Based on our experiments, the cause of the problem is the handrub trickling down inside the pump. Gravitational dispensers work fine, as flow back is not possible by design. Nevertheless, once the typical soft-shell container of a gravitational dispenser gets close to depletion, the dispensed amount may decrease radically. Regular dispensers can also work fine; the study identified regular dispensers that did not lose volume, even after 12 h. Dispensers work fine when filled with gel or soap, but can lose volume when filled with liquid handrub. That can be an explanation for the root of the problem, as most of these dispensers originally were designed to distribute soap. Our results suggest that most of the dispensers deliver less amount of gel than liquid ABHR (Fig. 7 and Fig. 10). As we read through the product descriptions of the investigated dispensers, we did not find different dispensed volumes documented for different product formats.

Figure 5 shows the provided volume after the dispensers were not used for various times. Notably, while dispensers were in continuous use, they dispensed 3 mL of ABHR following two consecutive pushes. If these dispensers were not in use for 2 h, then already 3 to 4 consecutive pushes were necessary to provide a total of 3 mL ABHR. This is alarming considering in particular that HCWs cannot objectively quantify the volume of handrub needed to clean their hands, and fail to notice that the dispenser aliquoted a smaller amount than usual (if so), leading to the risk of improper hand hygiene.

**Fig. 11:**
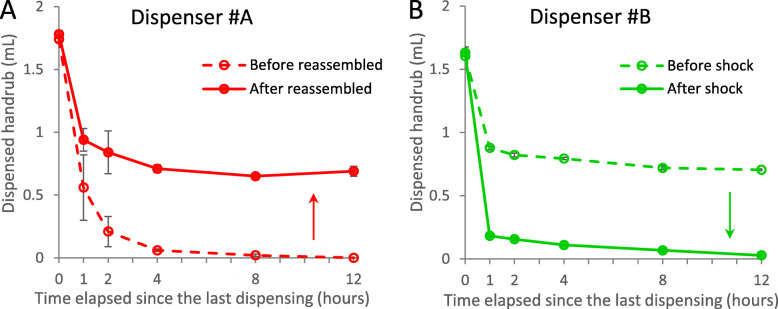
Maintenance can affect the performance of dispensers. **a**) Dispenser #A started to perform better after it was taken apart and reassembled. **b**) Dispenser #B started to perform worse after its stand fell during maintenance. Values are mean ± SD in 5 repeated measures (measurements actually showed very little deviation)

During our investigation, we noticed that after some physical impact, some dispensers started to operate differently. Dispenser #A was brand new when the measurements started. After it was taken apart, and carefully reassembled, it started performing better (Fig. 11a). This may raise questions of the production quality. Dispenser #B was also new, ordered directly for the conduct of the current study. After a few weeks of use, during regular maintenance, the dispenser stand fell, and while the dispenser did not crash, it took a heavy shock, and started to operate worse thereafter (Fig. 11b). Such a physical effect can occur at any time with a dispenser e.g., mounted on the wall at a hospital corridor, and as there were no external signs, the malfunction (degradation of dispensed volume) can go unnoticed. These results support that even the same type of dispensers can work differently, depending on their actual condition, underlining the importance of maintenance.

The study has limitations. Only dispensers from 5 manufacturers were tested. We tried to involve the most commonly used dispensers, but this can vary greatly by region, by country, or by hospitals. Unfortunately, we found limited information about what dispenser types are the most popular globally. We did not investigate precisely the age of the dispensers. New dispensers were marked at Table 3, but we did not have information about how old the used dispensers were. We also had no exact information about the age of the dispensers investigated in hospitals.

The volume-loss phenomenon was mentioned previously in a study performed in 1999. All handrub dispensers installed in a mid-size USA hospital were investigated. Among a total of 128 functioning dispensers (not broken, not empty, etc.), only 83 (65%) dispensed handrub from the first push; 17 dispensers (13%) had to be pushed twice to give some handrub while others had to be pushed three times (*n* = 11, 8.6%) or four or more times (*n* = 17, 13%) [11]. These findings correlate well with our data, while the cause of the problem was not further investigated in that study.

## Conclusions

Hand hygiene is a key integral part of multi-modal patient safety interventions. While significant research and development efforts have been invested in optimizing handrub composition, increasing staff compliance, and perfecting rubbing technique, other critical elements remained poorly tested and unexperimented. It is the case with the critical infrastructure of wall-mounted handrub dispensers that provide the vast majority of handrub supply in clinical settings across the world, in particular in high-resource countries. While the volume of the handrub dispensed arguably plays a critical role in the quality of hand antisepsis, adequate functioning of the dispensers has not been investigated before, to the best of our knowledge. There is no common regulation for handrub dispensers. Assadian et al. suggested standardized requirements the dispensers should meet [12]. Nevertheless, studying the time-dependency of the dispensed volume was not among the suggested tests.

The decrease in dispensed volume over time was found to be a generic phenomenon among commonly used dispensers. This study draws the attention to the criticality of hospital infrastructures, suggesting that handrub dispensers should be audited even before purchasing, and periodically thereafter. In the case of the worst dispensers (where dispensed volume decreases significantly already after 1 hour), the volume-loss can be detectable within minutes. Further investigations are needed to describe a reliable protocol for a rapid quality control test. Common type dispensers, especially when used with liquid handrub should be checked carefully.

Prior to the publication of this paper, the results of this study were brought to the attention of the World Health Organization’s POPS group (WHO Private Organizations for Patient Safety), where almost all major handrub manufacturers and dispenser manufacturers are represented. Further, the International Standardization Organization (ISO) TC 304 standards committee was notified, since they are working on a new global standard for hand hygiene (ISO DIS 23447) to include quality requirements with respect to wall-mounted dispensers as well.

## Data Availability

The datasets used and/or analyzed during the current study are available from the corresponding author on reasonable request.

## Abbreviations

ABHR: Alcohol-based handrub
HCW: Healthcare workers
WHO: Word Health Organization
